# Unmet needs for rehabilitation service of middle-aged and older adult residents in Chengdu, Sichuan, China: A cross-sectional study

**DOI:** 10.1038/s41598-023-38960-7

**Published:** 2023-07-25

**Authors:** Xichun Li, Yingxi Shi, Dan Zhao, Ke Jin, Jianmei Zhu, Ying Wang

**Affiliations:** 1 Department of Rehabilitation Medicine, Chengfei Hospital, Chengdu, 610073 People’s Republic of China; 2 Department of Medical Oncology, School of Medicine UESTC, Sichuan Academy of Medical Sciences & Sichuan Provincial People’s Hospital, Chengdu, 610072 People’s Republic of China

**Keywords:** Health care, Risk factors

## Abstract

To investigate the unmet needs for rehabilitation services among middle-aged and older adults in Chengdu, Sichuan, China, and identify the associated factors. This cross-sectional study was conducted on middle-aged and older adults in Chengdu, Sichuan, China, between 2015 and 2016. The questionnaire included demographic data and questions about rehabilitation needs. Multivariable logistic regression analysis was used to identify the associated factors of unmet needs for rehabilitation services. Among 663 participants, 91.70% needed medical rehabilitation (608/663), 26.55% of who need auxiliary equipment (176/663), 77.07% of who need daily care and social participation (511/663), and 79.34% of who need recreational therapy activities (526/663), while < 30% required auxiliary equipment. Multivariate logistic regression analysis showed that residents who were married, had annual income < CNY 80,000, had no medical insurance, had three or more health problems, were aged ≥ 60, and the disability status were independently associated with unmet needs for rehabilitation services (all *P* < 0.05). Marital status, annual income, medical insurance, health problems, and disability might be factors independently associated with the unmet needs for rehabilitation services. Attention should be paid to the financial burden of the population on rehabilitation services, and in addition to the disabled, the slow patients should also be given priority.

## Introduction

Many rehabilitation organizations, such as the International Society of Physical and Rehabilitation Medicine, have been formed in response to the recommendations of the United Nations and the World Health Organization for the active promotion of global rehabilitation medicine. This healthcare aspect in the People’s Republic of China has been associated with an increasing focus on improving the overall and long-term rehabilitation service. The status of rehabilitation medicine in China currently reflects the conclusions of existing studies on the need for rehabilitation services. China has established its three-tier service network, which has constantly been improving. However, despite the great demand for patients for rehabilitation services in Mainland China, these services, including community-based rehabilitation, seem inadequate. Nearly 90% of the older adults in Guangzhou are willing to receive rehabilitation treatment^1^. Community rehabilitation and home rehabilitation both have great demand and development potential in terms of physical function and subjective will of older adults^1^. Community schizophrenia patients have rehabilitation needs in many areas, including social security, emotional management, treatment compliance, and symptom management^2^. It is due to multiple reasons, such as high costs, lack of training in medical rehabilitation, and doubts related to the therapeutic benefits of rehabilitation^3^.

Previous studies on rehabilitation needs in China and other countries have concluded that it is important to consider the public’s perceptions in developing rehabilitation services^4^. Eldar et al.^5^ have demonstrated that China’s rehabilitation services are relatively closely aligned with clinical practice, while several previous studies reported a high need for rehabilitation services in Mainland China^1,6–9^.

One approach to learning about the need for rehabilitation services in communities is to study large samples of community populations. For example, Armstrong and colleagues^10^ studied the older adult population in Ontario, Canada, reporting that their needs for rehabilitation services were not effectively met^11^. These population-based studies are lacking in China, particularly those focusing on middle-aged or older adults.

Previous research has mainly focused on community groups with particular needs for rehabilitation services. For example, a study on the need for rehabilitation services among people with disabilities being discharged from hospitals in Poland revealed that orthopedic equipment and home renovation service did not sufficiently meet their needs^12^. Previous studies identified financial factors that could predict the use of rehabilitation resources, including physical dependence, major illness, time spent in medical care, having a family physician, having illnesses, age, and intact cognitive functions^13,14^. No similar study on rehabilitation needs in Mainland China has been conducted.

Therefore, this study investigated the need for rehabilitation services among all middle-aged and older adults in three Qingyang District communities in western Chengdu, the capital city of Sichuan Province, as well as the factors associated with the unmet needs for rehabilitation.

## Methods

### Study design and participants

This cross-sectional study investigated the unmet needs for rehabilitation services among middle-aged and older adults in three Chinese communities in Qingyang District, Chengdu, Sichuan, China, between 2015 and 2016. The inclusion criteria were (1) adults > 45 years with good cognitive ability, (2) permanent residents of Qingyang District, Chengdu, or outpatients, and (3) people with some need for rehabilitation. The exclusion criteria were (1) acute illnesses or (2) an incomplete questionnaire. This research was approved by The Chengfei Hospital Ethical Review Board (Approval number: 2014009; 1 September 2014). Written informed consent was obtained from all participants. All methods were carried out in accordance with relevant guidelines and regulations. All procedures were performed in accordance with the ethical standards laid down in the 1964 Declaration of Helsinki and its later amendments.

### Procedures

This study was based on a survey administered using a structured interview. The potential respondents were sent invitations in advance, saying that the investigators would contact them and that they were free to participate or not. Then, within 1 month of the invitation, the investigators met the respondents, obtained written informed consent, conducted the face-to-face interview, and filled in the interview table.

The determination procedures of the cognitive abilities of the participants were as follows. (1) Confirm whether participants had clear consciousness. The Glasgow Coma Scale (GCS) was used to determine the degree of consciousness disorder. Clear consciousness of the participants was a prerequisite for cognitive function assessment. (2) Screening for cognitive dysfunction. Under the condition of clear consciousness, the participants were screened for cognitive dysfunction using the Brief Psychiatric State Examination Scale (MMSE) and Cognitive Ability Examination Scale (CCSE), which is a key step in the assessment of cognitive dysfunction. The two scales are currently equally effective, and all participants completed both scales^15–22^. In the MMSE, a score < 24 is considered cognitive impairment^23^. In the CCSE, a score < 27 was considered cognitive impairment^24^. As long as a score on one of the scales meets cognitive dysfunction, it is considered cognitive dysfunction. (3) Those with intellectual disabilities were excluded from the sample according to the statistical principle of full consideration of the population sample.

### Sampling and sample size

At the end of 2014, the three communities had 69,771 residents, 10,933 of whom were > 45 years old. For sampling, this study assumed a response rate based on the usual response rate of questionnaire surveys in China, about 50%^25^, and sampled twice as many residents as necessary for stable statistical analysis. Thus, 820 residents were sampled using the simple random sampling (preliminary survey) and systematic sampling (formal survey) methods with SPSS. The respondents were randomly selected from the official residency lists.

### Questionnaire survey

In this study, the “need for rehabilitation” refers to any need that an individual with a health condition may have that requires rehabilitation management, interventions, and/or the use of specialized rehabilitation services or programs^26^. The “unmet needs for rehabilitation” that individuals might experience refer to any needs for rehabilitation services identified by a healthcare provider that the individual is unable to get due to different barriers^26^.

The City Rehabilitation Service Needs Questionnaire (CRNQ) includes questions about residents’ characteristics and rehabilitation needs. Appendix 1 presents the details of the CRNQ. Trained researchers, most of whom were professionals in rehabilitation medicine, were trained to administer the CRNQ. The first part of the CRNQ includes questions on the respondent’s demographic characteristics, health status, activity level, financial status, and social security characteristics. Disability was measured using the disability assessment questions of the Chinese Ministry of Health Rehabilitation Medicine teaching materials. Health status was measured using the first edition of the questionnaire on community rehabilitation needs for community rehabilitation science of the Ministry of Education of the People’s Republic of China and the National Health Commission of the People’s Republic of China. The second part of the CRNQ focuses on unmet needs for rehabilitation service, defined as “a service or a resource that would confer a health or rehabilitation gain”^27^, including medical rehabilitation, auxiliary equipment, daily care, social participation, and recreational therapy activities. At the end of the CRNQ, the respondents were asked to list all their unmet needs for rehabilitation services in order of importance.

A pilot study was conducted to ensure the questionnaire’s validity and reliability. A total of 30 middle-aged and older adults participated in the study. Based on discussions with the middle-aged and older adults who participated in the pilot study, categories were developed to help respondents understand the definitions of rehabilitation medicine and the need for rehabilitation services. After the preliminary survey, categories were developed to help respondents understand the definition of rehabilitation medicine and the need for rehabilitation services. During the questionnaire development process, the unclearness of the description was found for the questions of “family income” and “whether there is an elevator in the building” through the pre-survey, and then the questionnaire was revised after discussion with the expert group. Five experts in neurology, disability, public health, and rehabilitation medicine reviewed and approved the items. The experts found correlations between “service cost”, “home environment”, and “rehabilitation needs according to the research on rehabilitation needs” according to the following viewpoints. The unaffordability of the services, especially in low-income countries, represents an additional barrier for people in need of rehabilitation^26^. In addition, living in a residential home means better adjustment to the living environment and better provision of orthopedic and rehabilitation equipment^28^. Regarding reliability, the data used in the analysis were tested, and Cronbach’s reliability coefficient α was 0.92 for the sample (n = 663). The response rate was 80.8%.

### Disability

The definition of disability in the teaching materials of Rehabilitation Medicine of the Ministry of Health of China is “Disability refers to obvious physical and mental dysfunction caused by trauma, disease, developmental defects, or mental factors, affecting the ability to live, work, and learn normally to varying degrees. Disability in a broad sense includes sickness and disability, which is a general term for physical and mental dysfunction of the human body”^29^. The disability certificate from the Ministry of Civil Affairs was required to be provided by the respondents.

### Statistical analysis

SPSS version 23.0 software (IBM, Armonk, NY, USA) was used for statistical analysis. Chi-square for contingency or Fisher’s exact test was used for the categorical variables. Multivariable logistic regression analysis was used to estimate the odds ratios (ORs) and 95% confidence intervals (95% CI) of the relationships between independent variables and the perception of unmet needs for rehabilitation services. Only the statistically significant independent variables in the bivariable analysis were included in the multivariate regression model, and variables lost the significance in the multivariate analysis and were excluded from the final model and not shown. All *P* < 0.05 was considered statistical significance.

### Ethics approval

This research was approved by The Chengfei Hospital Ethical Review Board (Approval number: 2014009; 1 September 2014). Written informed consent was obtained from all participants. All methods were carried out in accordance with relevant guidelines and regulations. All procedures were performed in accordance with the ethical standards laid down in the 1964 Declaration of Helsinki and its later amendments.

## Results

There were 157 residents excluded: 145 did not complete the questionnaire (59 did not want to disclose their marital status, 72 did not want to disclose their financial status, and 14 did not want to disclose their health insurance), six had an acute stroke, and six reported no need for rehabilitation (Appendix 1). Therefore, the final sample consisted of 663 respondents, including 279 males and 384 females. There were 72.9% of respondents aged ≥ 60 years or older, 92.3% were married, 79.8% were without a college degree, and 67.4% reported annual income < CNY 80,000 per year. Regarding health, 90.3% had healthcare insurance, 1.7% reported disabilities, and 72.4% reported one or fewer health problems (Table 1).

**Table 1:** Basic characteristics (*n* = 663).

Variable	N (%)
Gender	
Male	279 (42.1)
Female	384 (57.9)
A)ge (in years	
< 60	180 (27.1)
> 60	483 (72.9)
Marital status	
Single	51 (7.7)
Married	612 (92.3)
Educational level	
Completed college	134 (20.2)
Less than college	529 (79.8)
Number of children	
0	8 (1.2)
1	285 (43.0)
2	181 (27.3)
3	112 (16.9)
≥ 4	77 (11.6)
Annual income (CNY/USD)^a^	
< 80,000/ 11,296	447 (67.4)
80,000–250,000/11,296–35,300	169 (25.5)
> 250,000/35,300	47 (7.1)
Residence	
Walk-up apartment	474 (71.5)
Bungalow or elevator apartment	189 (28.5)
Medical insurance	
Without	64 (9.7)
With	599 (90.3)
Disability	
Yes	11 (1.7)
No	652 (98.3)
Number of health problems^b^	
0	70 (10.6)
1	410 (61.8)
2	141 (21.3)
≥ 3	42 (6.3)

^a^CNY 1 = USD 0.1412, USD 1 = CNY 7.0805.

^b^Health problems include pain, dizziness, numbness, dysfunction.

Furthermore, regarding the unmet needs, the results showed that 91.70% (608/663) of the respondents had unmet needs for rehabilitation service, 26.55% (176/663) needed auxiliary equipment, 77.07% (511/663) needed daily care and social participation, and 79.34% (526/663) needed recreational therapy activities (Table 2).

**Table 2:** Variables that may have impact on unmet needs for rehabilitation service included (1) medical, (2) auxiliary (for walking or self-care or designated equipment for dressing, bathing and so on), (3) daily care and social participation, and (4) recreational therapy.

Variable	Medical rehabilitation^1^ (n = 608)	*P* value	Auxiliary equipment ^2^ (n = 176)	*P* value	Daily care and social participation^3^ (n = 511)	*P* value	Recreational therapy activities^4^ (n = 526)	*P* value
Gender								
Male	254 (91.0)	0.669	82 (29.4)	0.181	216 (77.4)	0.925	218 (78.1)	0.387
Female	354 (92.2)		94 (24.5)		295 (76.8)		308 (80.2)	
Age (years)								
< 60	164 (91.1)	0.752	15 (8.3)	*** < 0.001	127 (70.6)	*0.017	148 (82.2)	0.282
≥ 60	444 (91.9)		161 (33.3)		384 (79.5)		378 (78.3)	
Marital status								
Unmarried	43 (84.3)	*0.046	14 (27.5)	0.870	22 (43.1)	*** < 0.001	30 (58.8)	*** < 0.001
Married	565 (92.3)		162 (26.5)		489 (79.9)		496 (81.0)	
Education attainment (completed college)								
Yes	120 (89.6)	0.297	16 (11.9)	*** < 0.001	94 (70.1)	*0.038	114 (85.1)	0.073
No	488 (92.2)		160 (30.2)		417 (78.8)		412 (77.9)	
No. of children								
0	8 (100)	*0.022	0 (0)	*** < 0.001	6 (75)	0.197	7 (87.5)	*0.030
1	264 (92.6)		45 (15.8)		208 (73.0)		230 (80.7)	
2	156 (86.2)		50 (27.6)		141 (77.9)		135 (74.6)	
3	107 (95.5)		39 (34.8)		93 (83.0)		84 (75.0)	
≥ 4	73 (94.8)		42 (54.5)		63 (81.8)		70 (90.9)	
Annual income (CNY) ^a^								
< 80,000	415 (92.8)	**0.004	136 (30.4)	**0.003	350 (78.3)	*0.012	335 (79.0)	*** < 0.001
80–250,000	156 (92.3)		34 (20.1)		133 (78.7)		148 (87.6)	
> 250,000	37 (78.7)		6 (12.8)		28 (59.6)		43 (91.5)	
Residence								
Walk-up apartment	435(91.8)	0.920	129 (27.2)	0.560	373 (78.7)	0.117	371 (78.3)	0.290
Bungalow/elevator apartment	173(91.5)		47 (24.9)		138 (73.0)		155 (82.0)	
Medical insurance								
Without	52 (81.3)	**0.001	12 (18.8)	0.179	56 (87.5)	*0.041	44 (68.8)	*0.034
With	556 (92.8)		164 (27.4)		455 (76.0)		482 (80.5)	
Disability								
Yes	8 (72.7)	*0.021	7 (63.6)	*0.010	8 (72.7)	0.721	8 (72.7)	0.705
No	600 (52.0)		169 (25.9)		503 (77.1)		518 (79.4)	
No. of health * problems^b^								
0	55 (38.9)	*** < 0.001	11 (15.7)	0.142	51 (72.9)	*0.041	47 (67.1)	0.067
1	379 (39.0)		110 (26.8)		306 (74.6)		330 (80.5)	
2	133 (30.8)		43 (30.5)		121 (85.8)		115 (81.6)	
≥ 3	41 (97.6)		12 (28.6)		33 (78.6)		34 (81.0)	

*p < 0.05, **p < 0.01, ***p < 0.001.

^a^CNY 1 = USD 0.1412; USD 1 = CNY 7.0805.

^b^Health problems include pain, dizziness, numbness, dysfunction, etc.

The multivariable logistic regression analysis showed that participants who were married (OR = 2.365, 95% CI: 1.014 ~ 5.514, *P* = 0.046), had annual income < CNY 80,000 (OR = 1.610, 95% CI: 1.084 ~ 2.393, *P* = 0.018), were without medical insurance (OR = 2.568, 95% CI: 1.246 ~ 5.292, *P* = 0.011), and had three or more health problems (OR = 2.303, 95% CI: 1.446 ~ 3.664, *P* < 0.001) were more likely to have unmet medical rehabilitation needs. The factors that had a significant impact on unmet auxiliary equipment needs were age ≥ 60 (OR = 1.083, 95% CI: 1.062 ~ 1.104, *P* < 0.001) and disabilities (OR = 5.424, 95% CI: 1.403 ~ 20.968, *P* = 0.014). Unmet recreational needs were significantly higher among respondents who were married (OR = 2.902, 95% CI: 1.578 ~ 5.335, *P* = 0.001), had annual incomes < CNY 80,000 (OR = 2.168, 95% CI: 1.468 ~ 3.204, *P* < 0.001), or had no medical insurance (OR = 1.891, 95% CI: 1.051–3.403, *P* = 0.033) (Table 3). The other variables were not significant.

**Table 3:** Multivariable logistic regression analysis for the factors associated with the unmet medical rehabilitation needs.

Rehabilitation type	Variable	Odds ratio (OR)	95% Confidence Interval	*P* value
Medical rehabilitation	Marital status (Married versus Unmarried)	2.365	1.014–5.514	0.046
	Annual Income (< 80,000 versus > 80,000)	1.610	1.084–2.393	0.018
	Medical insurance (with versus without)	2.568	1.246–5.292	0.011
	Number of health problems (≥ 3 versus < 3)	2.303	1.446–3.664	< 0.001
Auxiliary equipment	Age (< 60 versus ≥ 60)	1.083	1.062–1.104	< 0.001
	Disability status (Yes versus No)	5.424	1.403–20.968	0.014
Recreational therapy activities	Marital status (Married versus Unmarried)	2.902	1.578–5.335	0.001
	Annual Income (< 80,000 versus > 80,000)	2.168	1.468–3.204	< 0.001
	Medical insurance (with versus without)	1.891	1.051–3.403	0.033

## Discussion

The present study showed that respondents who were married, had low income, or had health problems were more likely to report unmet needs for medical rehabilitation. Furthermore, having no medical insurance was associated with a higher possibility of reporting unmet medical and recreational rehabilitation needs. Importantly, the respondents aged ≥ 60 years old, particularly those who reported disability, had a significantly higher possibility of indicating unmet needs for auxiliary equipment.

These results are consistent with previous studies from other countries that reported how marital status influenced rehabilitation needs. In Sweden, the dyads, i.e., the social arrangement of two persons involved in an ongoing relationship or interaction, were found to influence rehabilitation service needs^30^. The study from Sweden revealed that patients’ caregivers estimated that the needs of those involved in an ongoing relationship or interaction 12 months after stroke were more likely to be met^30^. Another study found that marriage was related to a higher demand for medical rehabilitation^31^. The current study revealed that being married was associated with a higher possibility of reporting unmet medical rehabilitation needs, which contradicts Kołłątaj’s results in Poland^32^ that showed how the needs for rehabilitation services of unmarried individuals were relatively difficult to satisfy. With shorter hospital stays and more care and rehabilitation being delivered at home, the patient’s families and friends are likely to be more involved and have increased responsibilities for informal care and rehabilitation after, for example, a stroke. Studies showed that the people with whom someone shares their everyday life have an important role in the rehabilitation, and these people can prompt and urge the patients to seek rehabilitation^33–36^. Therefore, further research is needed to clarify the influences of marital status on the perception of rehabilitation service needs.

The relationship between income and rehabilitation service needs might be related to peoples’ decisions and ability to pay for service. People with little disposable income might hesitate to use costly services, delaying recovery or social integration after an illness. Consequently, the optimal period for recovery might pass without the provision of adequate intervention in rehabilitation, and some disabilities might become irreversible, thus further posing a burden on society^13^. According to Mitsch et al.^32^, rehabilitation after brain injury in Australia was associated with higher transportation and housing costs, putting a financial strain on family members and rehabilitation service providers. Hence, there is a relationship between medical service expenditures and rehabilitation service needs^32^. Yet, the previous studies mostly focused on people with particular disabilities, leaving a gap this study sought to overcome.

Several previous studies identified financial factors that could predict the use of rehabilitation resources^13,14^. The financial factors include the costs of the rehabilitation services corresponding to the family’s economic level. Kamenov et al.^26^ found that the unaffordability of the services, especially in low-income countries, represents an additional barrier for people in need of rehabilitation. The current study found that respondents without health insurance had more unmet rehabilitation needs than those with insurance. Consequently, an in-depth evaluation of medical insurance is required.

The respondents who reported more than three health problems were also perceived as having more unmet medical rehabilitation needs than healthier individuals, contrary to the findings of a previous study^5^. Older adults showed a low willingness to pay for a home-based rehabilitation service. Economic status and health conditions are the significant influencing factors of willingness to pay. The presence of diabetes or tumors positively influenced willingness to pay. Still, the reason for the difference remains unclear and needs to be further investigated. People without disabilities are thought to require fewer rehabilitation services from their caregivers than those with physical or cognitive disabilities^13^. The present study found that age and disability status influenced the possibility of reporting unmet rehabilitation service needs. The respondents aged ≥ 60 years were significantly more likely than the younger respondents to report unmet needs for auxiliary equipment (*P* < 0.001). Policymakers and healthcare professionals should adjust their efforts to ensure to meet the rehabilitation service needs of the older adults in the community. These findings are consistent with the results of Kołłątaj et al.^12^; nonetheless, they are still limited in Mainland China and have a limited application to individuals with disabilities^5^.

Some previous studies found correlations between educational attainment and rehabilitation service needs^37–39^. However, our results did not show any impact of education on unmet rehabilitation service needs; thus, further studies are needed to clarify the relationship.

The findings of the present study can assist in raising public awareness and education initiatives in the Rehabilitation Medicine Department of Chengfei Hospital and community health centers. This study may further the integration and guidance of management departments responsible for community rehabilitation resources. Moreover, it may further the efforts to increase medical insurance coverage and rehabilitation service projects, as well as improve facilities for people with disabilities.

Based on the obtained results, we would like to propose several public health recommendations: firstly, the government should establish rehabilitation quality control centers in the three-tier health system, secondary hospitals, and rehabilitation institutions, emphasizing the necessity to meet the needs of married people, those with low income, and the uninsured population in the community. Secondly, this study might serve as a valuable reference for the Community-Based Rehabilitation Committee of the Chengdu Society of Rehabilitation Medicine (a folk medicine organization) for its work in community rehabilitation. Thirdly, the reported data highlight the vulnerabilities of uninsured people, encouraging Chengdu Human Resource and Social Security Bureau to address these concerns.

Despite the aforementioned contributions, the current study has several limitations. First, the sample was not representative, considering the sampling was randomized in the three Qingyang District communities. The demographics of this city’s middle-aged and older population require further investigation. Nonetheless, this kind of community differentiation was used in previous studies^32^. Future studies should address these two demographic design flaws to provide a complete idea of the rehabilitation needs of older adults in Chengdu. Second, we examined fewer rehabilitation service types compared to previous studies in developed countries^40,41^. For example, we did not test unmet needs for physical therapy, occupational therapy, physiotherapy, traditional or basic rehabilitation, speech, swallowing, or psychological rehabilitation. Third, some social and financial factors were not tested. For example, a previous study in Nepal found that mountain geography, poor road infrastructure, and inconveniences influenced access to community rehabilitation services^42^. All our respondents were residents of urban Chengdu, located on the Chengdu Plain, where the altitude varies by no more than five meters. However, additional social and financial factors should be analyzed. Fourth, the rehabilitation service project implementers might have biased the research results^43^, which we did not control in this study. Finally, using established questions to assess rehabilitation service needs provides only general information. Future studies employing qualitative methods to understand better the needs for rehabilitation services and individual expectations for rehabilitation are required.

However, although similar studies have used different methods in other populations^44^, we administered questionnaires in person, considering that such an approach has been identified as the optimal approach in epidemiological research^45^.

## Conclusion

This study found that > 75% of residents had unmet needs for rehabilitation, including medical rehabilitation, daily care, social participation, and recreational therapeutic activities, while < 30% were in need of auxiliary equipment. Marital status, financial factors, and medical insurance affected the respondents’ rehabilitation service needs. Rehabilitation programs should target these vulnerable groups and acknowledge that the lack of auxiliary equipment for older adults and individuals with disabilities is a major problem in current rehabilitation efforts.

## Data Availability

All data generated or analyzed during this study are included in this published article.

## Appendix 1

**Table:**

Interview on rehabilitation needs of the elderly in the community
Rehabilitation coordinator_________ date_________ Hello! I am Chengfei Community Rehabilitation Coordinator_________, Thank you for allowing me to come to your home to complete this interview
i. personal information 1.Your full name: 2. Your gender: Male □ Female □ 3. Your age is ____ years old 4. Your marital status: (1). Unmarried □ (2). First marriage with spouse □ (3). Remarried with spouse □ (4). Divorce □ (5). Widowed □ 5. Have children: 1 Yes □ 2 No □ How many? _______ 6.Your address: 7. The type of your Residential: Building □ Elevator building □ Floor:______ 8.your telephone number: ______________ 9. Contact Name_______ Contact Phone______________ 10. Your family’s annual income: A. 80 thousands yuan or less □ B. 80 thousands -250 thousands yuan □ C. 250 thousands yuan or more □ (Family annual income is calculated at 40,000 yuan/person) 11. Do you have a disability certificate: 1. Yes □ 2. No □
ii. Your current disease situation1: 1. Cervical spondylosis □ 2. Lumbar disc herniation □ 3. Osteoarthritis □ 4. Bone hyperplasia □ 5. Shoulder periarthritis □ 6. Sequelae of cerebral infarction □ 7. Postoperative fracture □ 8. Brain injury □ (Fill in 2014 or 2015) 12. Your hospitalization diagnosis: (Complete the first diagnosis of the discharge certificate of the last hospitalization) 13. The main problems now: 1. Pain □ 2. Dizziness □ 3. Numb □ 4. Dysfunction □ 5. Other □ (fill in the current first complaint of the interviewe) 14.Time of discharge: 15. Have you received rehabilitation treatment at the hospital? : 1. Yes □ 2. No □ 16. What kind of treatment is it? 1. Acupuncture □ 2. Modality □ 3. Physical Therapy □ 4. Occupational therapy □ 5.Other therapy □ 17. Currently taking the drug:
III. Your health status and activity capacity before the onset (may be multiple) 18. Physical health: 1. High blood pressure □ 2. Diabetes □ 3. Coronary heart disease □ 4. Arthritis □ 5. Other □ 19. Self-care ability in life: 1 Unable to self-care □ 2 Basic self-care □ 3 Fully self-care □ 20. Activity ability (living condition): 1. Bedridden □ 2. Sitting at home □ 3. Walking at home □ 4. Moderate exercise □ 5. Very active □ 21. Communication with the outside world: 1. Rarely contact with outsiders □ 2. Want to communicate with others, but feel isolated. Can normal contact □ 3. More active □
IV. Your current health status and activity ability (multiple) 22. Physical health: 1. Hypertension □ 2. Diabetes □ 3.Coronary heart disease (CHD) □ 4. Hyperlipidemia □ 5. Angina pectoris □ 6. Heart failure □ 7. Myocardial infarction □ 8. Coronary bypass graft □ 9. After coronary stenting □ 10. Cerebral hemorrhage □11. Cerebral infarction □ 12. Fracture □ 13. Osteoporosis □ 14. Joint pain □ 15. Low back pain □ 16. Cervical spondylosis□ 17. Peripheral artery disease □18. Arthritis □19. Shit problem □ 20. Urine problem □ 21. Pressure sores □ 22. Language problem □ 23. Swallowing problems □ 24.other □ 23. Self-care ability in life: 1. Unable to self-care □ 2. Basic self-care □ 3. Fully self-care □ 24. Activity ability (living condition): 1.bedridden □ 2. Sitting at home □ 3. Walking at home □ 4. Moderate exercise □ 5. Very active □ 25. Communication with the outside world: 1. Rarely contact with outsiders □ 2. Want to communicate with others, but feel the gap □ 3. Able to communicate normally □ 4. Be active
v. Your current physical dysfunction: (Yes: 1; No: 2); muscle strength test /MMT: (0: A, 1: B, 2:C,3:D,4:E,5:F), Range of motion: Normal 3 decrease 2 abnormal 1 26.Upper limb: Arms: With or without atrophy□ MMT____ Range of motion_____ Hand: With or without atrophy□ MMT____ Range of motion_____ 27. Lower limbs: Legs: With or without atrophy □ MMT____ Range of motion_____ Foot: With or without atrophy □ MMT____ Range of motion_____ 28. Walking: 1.independent walking □ 2.with walking stick □ 3.wheelchair □ 4.bedridden □ 29. Self-care: 1. Completely dependent on others □ 2. Need help □ 3. Others prepared, their own □ 4. Finish independently □
vi. Participation in medical insurance and social security 30. 1.Chengfei internal medical insurance □ 2. Urban employees medical insurance □ 3.Urban residents medical insurance □ 4.Chengdu special outpatient service □ 5. New rural cooperative medical insurance □ 6. Other commercial medical insurance □ 7. Basic old-age insurance for urban workers □ 8. Basic old-age insurance for urban residents □ 9. New rural social old-age insurance □ 10. Not covered by any insurance
vii. What help do you hope to get in terms of rehabilitation? 31. Medical rehabilitation: 1. Acupuncture □ 2. Modality □ 3. Physical function recovery training □ 4. Self-care skills training □ 5. Chinese herb □ 6. Speech function training □ 7.Swallowing function training □ 8.Psychological counseling □ 9.Other □ (rehabilitation treatment, Modality, Physical Therapy, Occupational Therapy, TCM rehabilitation therapy, speech therapy, swallowing therapy, psychological therapy, etc.) 32. Auxiliary equipment: 1 .Walking stick □ 2. Wheelchair □ 3. Home environment improvement □ (may be ramp, toilet seat, door width or kitchen counter) 33. Daily care and social participation: 1. Life care □ 2. Out □ 3. Economic assistance □ 4. Other □ 34. Recreational Therapy Needs: 1. To participate in cultural activities (including square dance) □ 2. To participate in sports training □ 3. Training venue □ 4. Training equipment □ 5. To participate in sports competition □ 6. Guidance coach □ 7. Art guidance teacher □ 8. Activity venue □ 9. Audio-visual reading materials □ 10. Cultural supplies □ 35. Your evaluation of our department of rehabilitation medicine in chengfei hospital Average daily cost: 1.200–400 □ 2.400–600□ 3.600–800□ Facilities and equipment: 1. Complete □ 2. Basic meet □ 3. Lack more □ Service attitude: 1. Excellent □ 2. Good □ 3. Poor □ Treatment effect: 1. Improvement □ 2. Remission □ 3. Poor □

## References

[1] Luo X, Yang Q, Huang L, Luo J, Chen Z, Zhao H, Liu H (2022). Investigation and analysis of community and home rehabilitation needs of the elderly in Guangzhou. Chin. J. Rehab. Med..

[2] Ge C, Zhang W, Yang L, Zhang Q, Cai J (2019). Study on rehabilitation needs and influencing factors of community schizophrenia patients in Shanghai. Chin. J. Gen. Pract..

[3] Asakawa T, Zong L, Wang L, Xia Y, Namba H (2017). Unmet challenges for rehabilitation after stroke in China. Lancet.

[4] Dai H, Xue H, Yin ZJ, Xiao ZX (2006). The need and its influence factors for community-based rehabilitation services for disabled persons in one district in Beijing. Biomed. Environ. Sci..

[5] Eldar R, Kullmann L, Marincek C, Sekelj-Kauzlarić K, Svestkova O, Palat M (2008). Rehabilitation medicine in countries of Central/Eastern Europe. Disabil. Rehabil..

[6] Organization WH: Global Burden of Disease (GBD) Disease and injury country estimates for 2004 by cause for WHO member States.

[7] WHOatW. B: World report on disability. . *Geneva, Switzerland: WHO* 2011.

[8] Sheng WW, Li X, Qiu ZY, Qang GX, Li L, Sun HW, Shen ZH, Chen JN, Ma HZ, Li AQ (2020). Research on the rehabilitation needs of disabled children and the development of rehabilitation services. China Rehab. Theory Pract..

[9] Yang L (2019). Investigation and Analysis of Rehabilitation Needs of Patients with Schizophrenia and Their Caregivers in Community.

[10] Kolominsky-Rabas PL, Weber M, Gefeller O, Neundoerfer B, Heuschmann PU (2001). Epidemiology of ischemic stroke subtypes according to TOAST criteria: Incidence, recurrence, and long-term survival in ischemic stroke subtypes: A population-based study. Stroke.

[11] Wang Y, Levi CR, Attia JR, D’Este CA, Spratt N, Fisher J (2003). Seasonal variation in stroke in the Hunter Region, Australia: a 5-year hospital-based study, 1995–2000. Stroke.

[12] Armstrong JJ, Zhu M, Hirdes JP, Stolee P (2015). Rehabilitation therapies for older clients of the Ontario home care system: Regional variation and client-level predictors of service provision. Disabil. Rehabil..

[13] Chang YY, Peng LN, Lin MH, Lai HY, Chen LK, Hwang SJ, Lan CF (2011). Who determines the rehabilitation needs of care home residents?. Obs. Survey. Arch. Gerontol. Geriatr..

[14] Lin JD, Yen CF, Loh CH, Li CW, Wu JL (2006). Rehabilitation service utilization and determinants among people with an intellectual disability: preliminary findings in Taiwan. Disabil. Rehabil..

[15] National Health and Family Planning Commission: *Rehabilitation Function Assessment: The 13th Five-Year Plan.* Beijing: National Health and Family Planning Commission.

[16] Meyer JS, Rauch GM, Crawford K, Rauch RA, Konno S, Akiyama H, Terayama Y, Haque A (1999). Risk factors accelerating cerebral degenerative changes, cognitive decline and dementia. Int. J. Geriatr. Psychiat..

[17] Folstein MF, Folstein SE, McHigh PR (1975). A practical method for grading the cognitive state of patients for the clinician. J. Psychiatr. Res..

[18] Jacobs JW, Bernhard MR, Delgado A, Strain JJ (1977). Screening for organic mental syndromes in the medically ill. Ann. Intern. Med..

[19] Kaufman DM, Weinberger M, Strain JJ, Jacobs JW (1979). Detection of cognitive deficits by a brief mental status examination: The cognitive capacity screening examination, a reappraisal and a review. Gen. Hosp. Psychiat..

[20] Grigoletto F, Zappala G, Anderson DW, Lebowitz BD (1999). Norms for the mini-mental state examination in a healthy population. Neurology.

[21] Meyer JS, Judd BW, Tawaklna T, Rogers RL, Mortel KF (1986). Improved cognition after control of risk factors for multi-infarct dementia. JAMA.

[22] Meyer JS, Thornby J, Crawford K, Rauch GM (2000). Reversible cognitive decline accompanies migraine and cluster headaches. Headache.

[23] Pezzotti P, Scalmana S, Mastromattei A, Di Lallo D (2008). Progetto Alzheimer working G: The accuracy of the MMSE in detecting cognitive impairment when administered by general practitioners: a prospective observational study. BMC Fam. Pract..

[24] Meyer JS, Li YS, Thornby J (2001). Validating mini-mental status, cognitive capacity screening and Hamilton depression scales utilizing subjects with vascular headaches. Int. J. Geriatr. Psychiat..

[25] Weiqun S, Weifang F, Shaoxia F (2018). Practice of quality control circle activity to improve the response rate of discharge questionnaire in follow-up system. Huangzhou Pharm..

[26] Kamenov K, Mills JA, Chatterji S, Cieza A (2019). Needs and unmet needs for rehabilitation services: A scoping review. Disabil. Rehabil..

[27] Kołłątaj B, Gorczyca R, Kołłątaj W, Jędrych M, Sobieszczańska A, Sobieszczański J, Karwat ID (2015). Meeting needs for rehabilitation equipment and home adjustments among the disabled in their life environment. Ann. Agric. Environ. Med..

[28] Kollataj B, Gorczyca R, Kollataj W, Jedrych M, Sobieszczanska A, Sobieszczanski J, Karwat ID (2015). Meeting needs for rehabilitation equipment and home adjustments among the disabled in their life environment. Ann. Agric. Environ. Med..

[29] Huang X, Yan T (2013). Rehabilitation Medicine.

[30] Ullberg T, Zia E, Petersson J, Norrving B (2016). Perceived unmet rehabilitation needs 1 year after stroke: an observational study from the swedish stroke register. Stroke.

[31] Ekstam L, Johansson U, Guidetti S, Eriksson G, Ytterberg C (2015). The combined perceptions of people with stroke and their carers regarding rehabilitation needs 1 year after stroke: A mixed methods study. BMJ Open.

[32] Mitsch V, Curtin M, Badge H (2014). The provision of brain injury rehabilitation services for people living in rural and remote new South Wales. Australia. Brain Inj..

[33] Ekstam L, Tham K, Borell L (2011). Couples’ approaches to changes in everyday life during the first year after stroke. Scand. J. Occup. Ther..

[34] Jongbloed L (1994). Adaptation to a stroke: The experience of one couple. Am. J. Occup. Ther..

[35] Robinson-Smith G, Mahoney C (1995). Coping and marital equilibrium after stroke. J. Neurosci. Nurs..

[36] van Nes F, Runge U, Jonsson H (2009). One body, three hands and two minds: A case study of the interwined occupations of an older couple after a stroke. J. Occup. Sci..

[37] Szybalska A, Broczek K, Slusarczyk P, Kozdron E, Chudek J, Puzianowska-Kuznicka M, Kostka T, Skalska A, Mossakowska M (2018). Utilization of medical rehabilitation services among older Poles: Results of the PolSenior study. Eur. Geriatr. Med..

[38] Gell NM, Mroz TM, Patel KV (2017). Rehabilitation services use and patient-reported outcomes among older adults in the United States. Arch. Phys. Med. Rehabil..

[39] Moreland BL, Durbin LL, Kasper JD, Mielenz TJ (2018). Rehabilitation utilization for falls among community-dwelling older adults in the United States in the national health and aging trends study. Arch. Phys. Med. Rehabil..

[40] Thorsen L, Gjerset GM, Loge JH, Kiserud CE, Skovlund E, Fløtten T, Fosså SD (2011). Cancer patients’ needs for rehabilitation services. Acta Oncol..

[41] Gell NM, Patel KV (2019). Rehabilitation services use of older adults according to fall-risk screening guidelines. J. Am. Geriatr. Soc..

[42] Blanchet K, Girois S, Urseau I, Smerdon C, Drouet Y, Jama A (2014). Physical rehabilitation in post-conflict settings: Analysis of public policy and stakeholder networks. Disabil. Rehabil..

[43] Fernandez RS, Davidson P, Griffiths R, Salamonson Y (2011). Improving cardiac rehabilitation services–challenges for cardiac rehabilitation coordinators. Eur. J. Cardiovasc. Nurs..

[44] Kersten P, McLellan DL, Gross-Paju K, Grigoriadis N, Bencivenga R, Beneton C, Charlier M, Ketelaer P, Thompson AJ (2000). A questionnaire assessment of unmet needs for rehabilitation services and resources for people with multiple sclerosis: Results of a pilot survey in five European countries. Needs task group of MARCH (Multiple sclerosis and rehabilitation, care and health services research in Europe). Clin. Rehabil..

[45] Bowling A (2005). Mode of questionnaire administration can have serious effects on data quality. J. Public Health Oxf..

